# Cerebellar Hemangioblastoma Mimicking Arteriovenous Malformation: A Case Report

**DOI:** 10.7759/cureus.60671

**Published:** 2024-05-20

**Authors:** Abdullah A Al-Mutairi, Abdulkhaliq AlHifzi, Rinad Alghoraiby, Talal Faden

**Affiliations:** 1 Diagnostic Radiology, Prince Sultan Military Medical City, Riyadh, SAU; 2 Diagnostic Radiology, King Khalid University Hospital, Riyadh, SAU

**Keywords:** neuroradiology, imaging, avm, hemangioblastoma, case report

## Abstract

Hemangioblastoma (HBM) is a tumor distinguished by the presence of stromal cells and small vessels. These stromal cells represent stem cells, which, due to the influence of the neoplasm, proliferate and differentiate into "vasoformative elements" that create new blood vessels.

Hemangioblastomas resemble arteriovenous malformation (AVM) in imaging features, characterized by an apparent vascular blush, the presence of multiple feeding vessels, and evident draining veins observed on digital subtraction angiography (DSA). Our study presents a case of HBM in the right cerebellar hemisphere mimicking AVM. The patient had been diagnosed with AVM in the same location two years ago and managed with endovascular embolization. One month prior, the patient experienced severe headaches, imbalance, nausea, left ear fullness, blurry vision, and high blood pressure. The imaging feature suggests HBM rather than AVM. The patient next underwent sub-occipital craniotomy and tumor resection with external ventricular drainage (EVD) insertion. The histopathological report of the excised mass confirmed HBM.

In conclusion, AVM and HBM rarely occur together. Recent research indicates that HBM and AVM have exact embryologic origins and need later genetic alterations to develop into symptomatic lesions. Further research is required to clarify the uncommon combination of these lesions.

## Introduction

The term "hemangioblastoma (HBM)" was first proposed by Cushing and Bailey in 1928 and refers to benign cystic and/or solid neoplasms rich in vasculature in the central nervous system. Hemangioblastomas account for approximately 1%-2.5% of all intracranial tumors and 2%-3% of all intramedullary neoplasms. The cerebellum is the most common location, but it can also occur beyond the confines of the central nervous system [1].

The incidence of HBM peaks between the third and fifth decades of life, and HBM is more common in males (1.3:1 ratio) [2]. HBM most frequently occurs as sporadic lesions. However, around 20% of HBM lesions are associated with Von Hippel-Lindau syndrome. Clinical manifestations of HBM are nonspecific and include headache, cerebellar dysfunction, and an altered mental state [3]. The gold-standard treatment is surgical resection of symptomatic tumors when the procedure's benefits outweigh the surgical risks [3].

Magnetic resonance imaging (MRI) is most commonly used to diagnose HBM. The solid part of HBM manifests as iso- or hypointense on T1-weighted images, hyperintense on T2-weighted images, and a significant contrast enhancement on T1 post-contrast [4]. Cystic hemangioblastoma resembles arteriovenous malformation (AVM) in imaging features, characterized by an apparent vascular blush, the presence of multiple feeding vessels, and evident draining veins observed on digital subtraction angiography (DSA) [4].

The present study reports a case of cerebellar HBM that was diagnosed as AVM and treated with endovascular embolization.

## Case presentation

A 28-year-old man was admitted to the emergency department at King Khalid University Hospital in Riyadh. He had been diagnosed with AVM two years ago and managed with endovascular embolization. He had been in his usual state of health until one month ago when he started to experience severe headaches, imbalance, nausea, left ear fullness, blurry vision, and high blood pressure. He denied losing consciousness, seizures, or abnormal movement; a review of his systems was unremarkable. His headache and imbalance kept worsening. Upon examination, he was vitally stable except for blood pressure: 155/100. His Glasgow coma scale score was 15/15 (eye response 4, verbal response 5, motor response 6); on the finger-to-nose test, he overshot on the left side. On the heel-to-shin test, he could not run his heel down to his shin; he exhibited a zig-zagging movement on the left side. An ophthalmological examination revealed disk swelling, Frisen grade 1 on the right eye, and Frisen grade 2 on the left eye.

**Figure 1:**
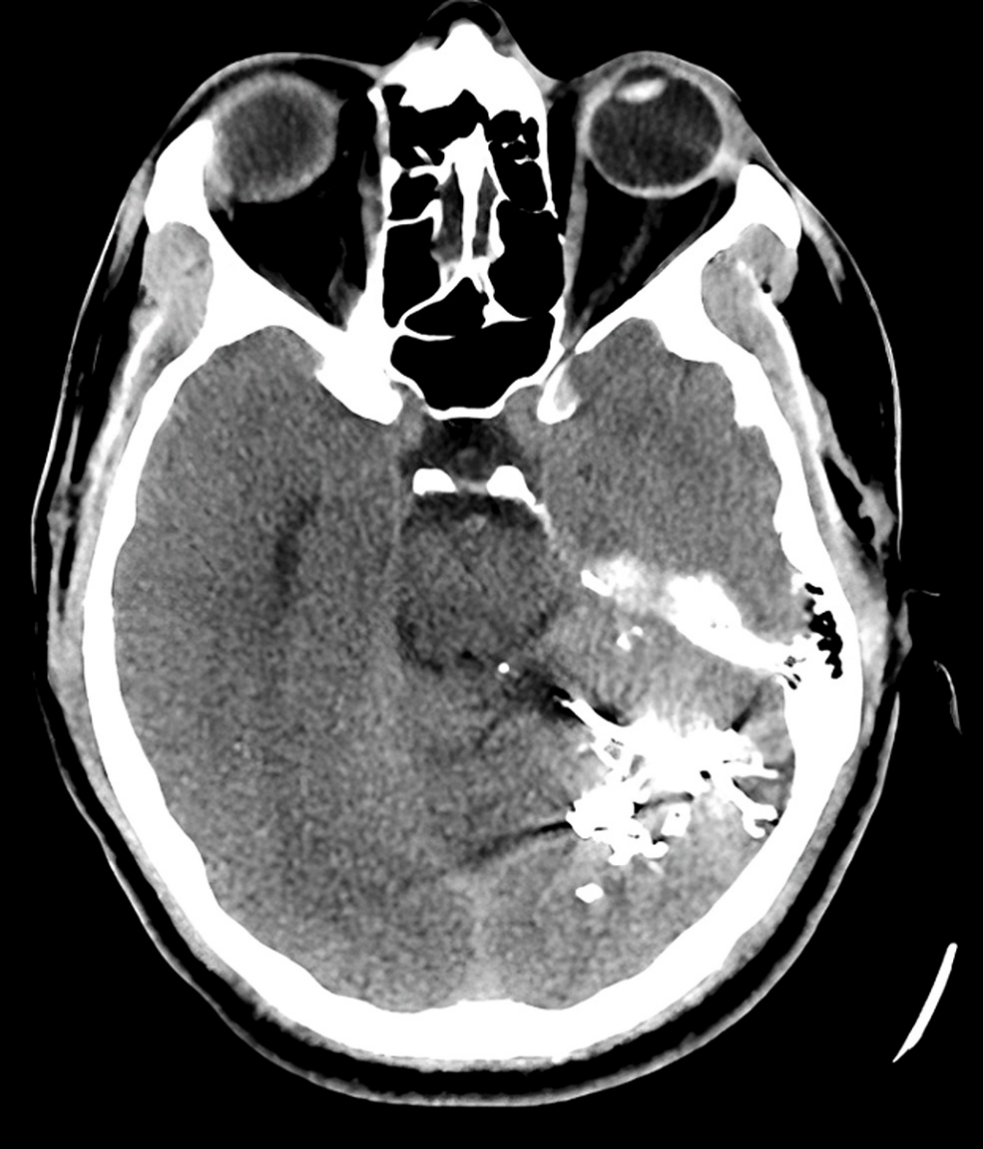
Post-embolization CT brain without contrast

The patient's total laboratory and renal function tests were unremarkable. An electrocardiogram revealed sinus rhythm with no ischemic changes. Two years before, a post-embolization CT brain without contrast showed post-embolization changes without signs of cystic mass (Figure 1).

**Figure 2:**
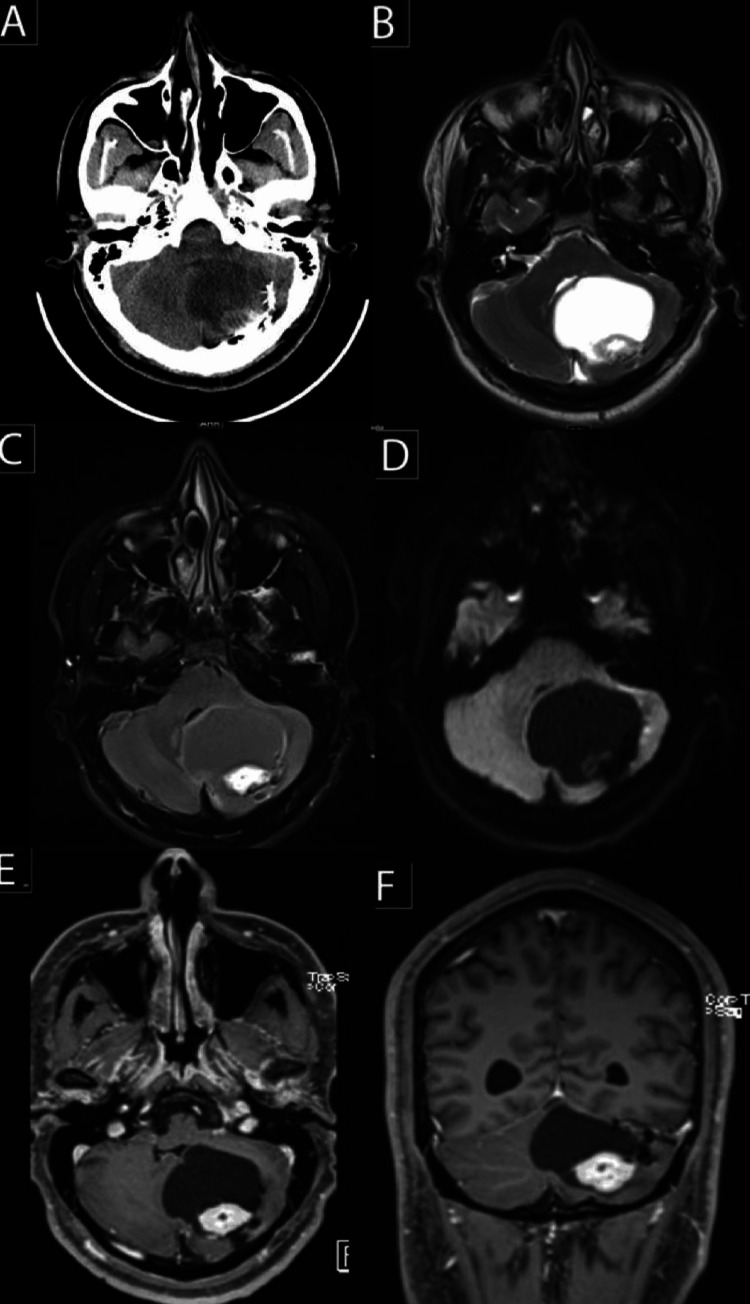
(A) CT brain without contrast. (B) T2-weighted image. (C) FLAIR. (D) Diffusion-weighted image. (E) Axial T1 post-contrast. (F) Coronal T1 post-contrast. FLAIR: Fluid-attenuated inversion recovery.

At the time of presentation, an unenhanced CT scan of the brain revealed a large cystic lesion in the left cerebellar hemisphere measuring 5.2 x 4.0 x 3.8 cm; it exerted a marked mass effect on the fourth ventricle and cerebellar hemispheres and was associated with mild dilatation of the lateral ventricles. A brain MRI revealed a large complex left posterior fossa mass measuring 5.8 x 5.0 x 4.6 cm with a medium-sized eccentric enhanced component measuring 2.6 x 1.7 x 1.9 cm (Figure 2).

The patient underwent both an abdominal CT scan and an MRI of the spine to rule out any additional lesions indicating Von Hippel-Lindau syndrome. Those investigations were all unremarkable.

**Figure 3:**
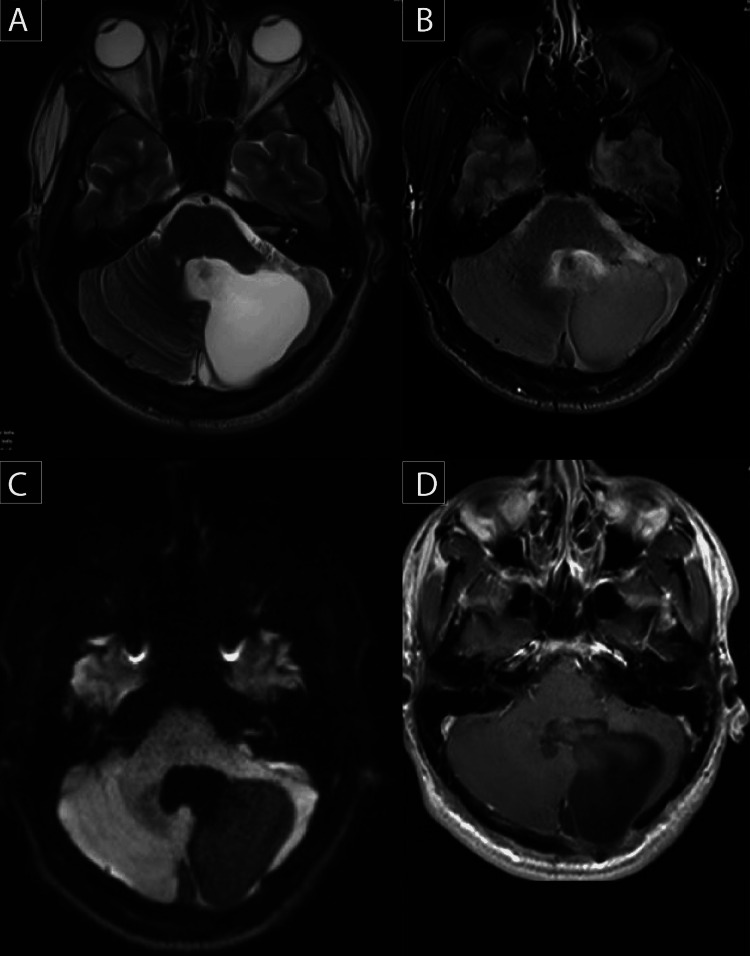
(A) T2-weighted image. (B) FLAIR. (C) Diffusion-weighted image. (D) Axial T1 post-contrast. FLAIR: Fluid-attenuated inversion recovery.

The patient next underwent sub-occipital craniotomy and tumor resection with external ventricular drainage (EVD) insertion. The histopathological report of the excised mass confirmed HBM. Eight months post-surgical imaging, an MRI revealed a complete resection of the left cerebellar hemisphere complex mass without a soft tissue component (Figure 3).

## Discussion

HBM is a tumor distinguished by the presence of stromal cells and small vessels. These stromal cells represent stem cells, which, due to the influence of the neoplasm, proliferate and differentiate into "vasoformative elements" that create new blood vessels. However, vascular abnormalities of HBM can mimic AVM [5]. AVMs have historically been considered congenital. They are either sporadic or syndromic in origin. Angioblasts differentiate from mesoderm during the third week of embryonic development, forming arterial, venous, and capillary vessels [6]. An AVM is a direct abnormal connection between arteries and veins, lacking intervening capillaries [7]. One study reported 74 cases of brain tumors associated with AVM [8]. Oligodendroglioma was the most common glial mass associated with AVM [8].

Recent research indicates that hemangioblastomas in Von Hippel-Lindau disease may originate during embryonic development [3]. The hemangioblasts, which serve as embryonic precursors of hematopoietic and endothelial cells, are the center point of this process [5]. The hemangioblast may arrest but can subsequently become reactivated, leading to the formation of HBM [9]. One study reported that tumor growth may be slow and remain undetectable for years on imaging [9]. However, most embryological studies of HBM have focused on tumors linked to Von Hippel-Lindau disease [9]. Therefore, these findings may not necessarily apply to sporadic HBM.

A study has reported that their shared embryologic origin might explain the coexistence of AVM and HBM [10]. Furthermore, another study proposed that HBM and AVM might share an embryologic origin but undergo subsequent genetic alterations [11]. The patient presented with AVM and was managed with endovascular embolization outside our institution. Two years later, the patient presented with the imaging feature of HBM in the same location. It is possible that HBM was coexistent with the AVM at the time of diagnosis; however, there are no pre-embolization images to confirm the coexistence of HBM and AVM.

## Conclusions

The patient presented with an AVM and was managed with endovascular embolization. However, two years later, we also found an imaging feature indicative of HBM in the same location. HBM coexisting with the AVM at the time of diagnosis cannot be excluded. Recent research indicates that HBM and AVM have exact embryologic origins and need later genetic alterations to develop into symptomatic lesions. Further research is required to clarify the uncommon combination of these lesions.
